# The detrimental effects of radiotherapy interruption on local control after concurrent chemoradiotherapy for advanced T-stage nasopharyngeal carcinoma: an observational, prospective analysis

**DOI:** 10.1186/s12885-018-4495-2

**Published:** 2018-07-16

**Authors:** Ji-Jin Yao, Ya-Nan Jin, Si-Yang Wang, Fan Zhang, Guan-Qun Zhou, Wang-Jian Zhang, Zhi-Bin, Cheng, Jun Ma, Zhen-Yu Qi, Ying Sun

**Affiliations:** 1 Department of Radiation Oncology, Sun Yat-sen University Cancer Center, State Key Laboratory of Oncology in South China, Collaborative Innovation Center for Cancer Medicine, Guangzhou, 510060 Guangdong Province People’s Republic of China; 2 Department of Radiation Oncology, the Fifth Affiliated Hospital of Sun Yat-sen University, Zhuhai, 519001 Guangdong Province China; 3 Department of Medical Statistics and Epidemiology & Health Information Research Center & Guangdong Key Laboratory of Medicine, School of Public Health, Sun Yat-sen University, Guangzhou, 510080 Guangdong Province China

**Keywords:** Nasopharyngeal carcinoma, Radiotherapy interruption, Local control, Concurrent chemoradiotherapy, Advanced T stage

## Abstract

**Background:**

Previous studies have reported radiotherapy interruption (RTI) is associated with poor local control in two-dimensional radiotherapy (2DRT) era. However, it remains unclear whether RTI still affects local control for advanced T stage (T3–4) in the intensity-modulated radiation therapy (IMRT) era. We aim to evaluate whether RTI affects local control for T3–4 NPC treated with definitive IMRT.

**Methods:**

In this observational prospective study, 447 T3–4 NPC patients treated with IMRT plus concurrent chemotherapy were included. All patients completed the planned radiotherapy course, and RTI was defined as the actual time taken to finish the prescribed course of radiotherapy minus the planned radiotherapy time. Receiver operating characteristic (ROC) curve was used for determined the cutoff point of RTI. The effects of RTI on local control were analyzed in multivariate analysis.

**Results:**

At 5 years, the local relapse-free survival (LRFS) and overall survival (OS) rates were 93.7 and 85.7%, respectively. The cutoff RTI for LRFS was 5.5 days by ROC curve. Compared to patients with RTI >  5 days, patients with RTI ≤ 5 days had a significantly lower rate of LRFS (97% vs. 83%; *P* < 0.001). In multivariate analysis, RTI was a risk factor independently associated with LRFS (HR = 9.64, 95% CI, 4.10–22.65), but not for OS (HR = 1.09, 95% CI, 0.84–1.64).

**Conclusions:**

The current analysis demonstrates a significant correlation between prolonged RTI and local control in NPC, even when concurrent chemotherapy is used. We consider that attention to RTI seems to be warranted for patients with advanced T-stage NPC in the era of IMRT.

**Electronic supplementary material:**

The online version of this article (10.1186/s12885-018-4495-2) contains supplementary material, which is available to authorized users.

## Background

In Southern China, nasopharyngeal carcinoma (NPC) is a common malignancy [1, 2]. Radiotherapy is the mainstay of treatment of NPC given the anatomical restrictions and its radio-sensitivity [3]. The tumor often present with bulky disease and located near multiple critical structures, leading to difficulties in achieving satisfactory local control using two dimensional radiotherapy. Several studies have reported a 5-year local relapse-free survival (LRFS) of 61–79% and overall survival (OS) of 59–69% using two dimensional radiotherapy [4, 5].

With advances in radiation technology, intensity-modulated radiotherapy (IMRT) has become the primary means of radiotherapy due to better treatment outcome. The phase II trial of RTOG 0225 conducted by Memorial Sloan-Kettering Cancer Center reported the excellent local control (2-year rate, 92.6%) for NPC in the era of IMRT [6]. Additionally, Peng et al. [7] conducted a randomised study and found that IMRT had a significant improvement in local control of 7.7% (5-year rate) compared with two dimensional radiotherapy. However, approximately 8–10% patients still experience local relapse in the era of IMRT, which has become a major cause of treatment failure in NPC [8, 9].

Many prognostic factors may directly and/or indirectly affect the local control, including radiotherapy interruption (RTI), which is a significant independent factor in the local control of lung cancer [10], laryngeal cancer [11] and NPC [12, 13] using two dimensional radiotherapy. However, it remains unknown whether RTI still affects local control in the era of IMRT. Based on this knowledge, we, therefore, did an observational prospective study to identify the relationship between RTI and local control in patients with stage T3–4 stage NPC treated by definitive IMRT.

## Methods

### Patient characteristics

Between December 2009 and February 2012, we included a total of 447 NPC patients. Patients’ characteristics are listed in Additional file 1: Table S1. The eligibility criteria were as follows: (1) histologically proven NPC, (2) stage with T3 to T4, (3) no evidence of distant metastases, (4) treated by IMRT and finished the planned radiotherapy, (5) received concurrent chemotherapy, and (6) no prior history of malignancy. Patients were staged based on American Joint Committee on Cancer (AJCC) staging system (7th edition, 2009) [14]. This study was approved by our center’s Institutional Review Board. The authenticity of this article has been validated by uploading the key raw data onto the Research Data Deposit public platform (www.researchdata. org.cn), and the approval Research Data Deposit number is RDDB2018000277.

### Radiotherapy and chemotherapy

IMRT was administered to all patients included in the study. We delineated the target volumes using a previously described treatment protocol by Sun Yat-sen University Cancer Center [15], which is consistent with International Commission on Radiation Units (ICRU) and Measurements reports 62 [16] and 83 [17]. All patients received concurrent chemotherapy, which consisted of 80–100 mg/m^2^ cisplatin every 3 weeks or 40 mg/m^2^ weekly. Deviations from these guidelines were due to patient refusal or when organ dysfunction suggested intolerance to chemotherapy.

### The definition of RTI

Radiation treatment time was calculated as the duration from start of radiotherapy to completion of the planned course. All patients were treated with a fraction daily for 5 days per week, and no planned interruption. Radiotherapy interruptions were allowed in the case of holidays, machinery faults, severe acute toxicity, and other causes. RTI was defined as the radiation treatment time minus the planned radiation time (assuming a Monday start).

### Follow-up

During treatment, patients were observed at least one time a week. After treatment, patients were then evaluated once every 3 months in the first three years, once every 6 months for the following two years, and once every afterward. The end points contained LRFS and OS. We defined LRFS from the date of initial treatment to the date of the first nasopharynx recurrence; and OS was calculated from the date of initial treatment to death. Local relapses were diagnosed by biopsy, MRI, or both.

### Statistical analysis

Receiver operating characteristic (ROC) curves were used to determine the RTI cutoff point for LRFS. Chi-square test was used to determine the differences in patients’ characteristics among groups. Survival rates were depicted by Kaplan–Meier curves and were compared by Log-rank tests. A Cox proportional hazards model was used to test the significant factors in multivariate analysis. A two-tailed *P* value < 0.05 was deemed statistically significant. We performed all analyses using R 3.1.2 software.

## Results

### Patient characteristics

**Fig. 1:**
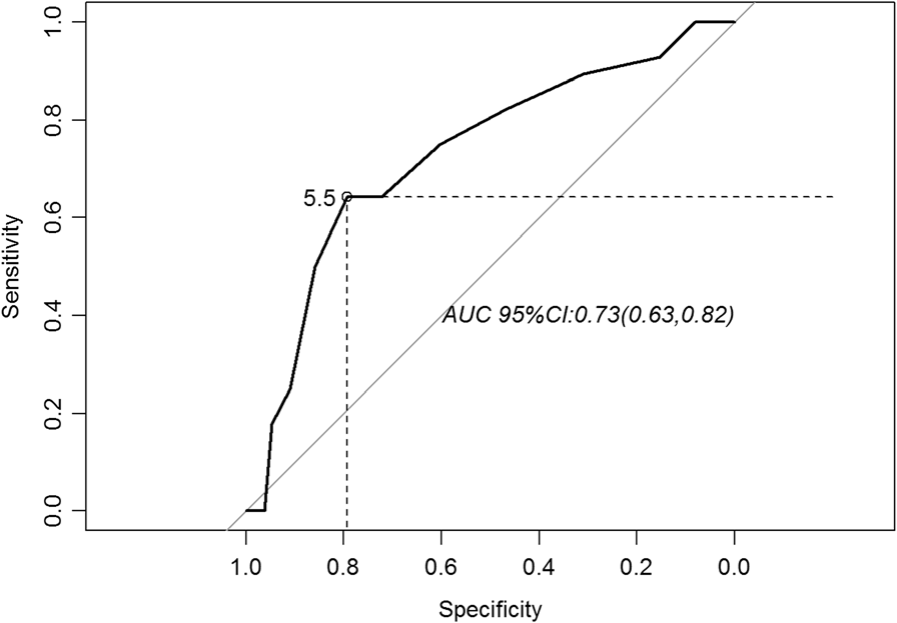
Receiver operating characteristic (ROC) curve analysis showing the effect of RTI on locally advanced NPC with respect to LRFS

The ability of RTI to predict LRFS was shown by ROC curve (Fig. 1), and the best RTI cutoff for LRFS was 5.5 days (area 0.73; 95% CI, 0.63–0.82). Based on optimal cutoff point, all patients were divided into RTI ≤ 5 days group or RTI >  5 days group. The baseline characteristics of the two groups are listed in Table 1. There were no differences in terms of age, sex, pathologic features, T (tumor) stage, N (nodal) stage, overall stage or schedule dose (all *P* > 0.05). However, patients receiving a schedule dose of 70 Gy in 33 fractions (2.12 Gy/F) were significantly (*P* = 0.013) more likely to have a longer RTI (> 5 days) than patients who received a dose of 68 Gy in 30 F (2.27 Gy/F).

**Table 1:** Patient and tumor characteristics

Characteristic	RTI ≤ 5 days (*n* = 342)	RTI > 5 days (*n* = 105)	*P*-value^*^
	No. of patients (%)	No. of patients (%)	
Age (years)			0.168
≤ 50	221 (64.6)	60 (57.1)	
> 50	121 (35.4)	45 (42.9)	
Sex			0.699
Male	260 (76)	78 (74.3)	
Female	82 (24)	27 (25.7)	
Pathology			0.877
I	2 (0.6)	0 (0)	
II	15 (4.4)	5 (4.8)	
III	325 (95)	100 (95.2)	
T stage^a^			0.775
T3	277 (81)	87 (82.9)	
T4	65 (19)	18 (17.1)	
N stage^a^			0.416
N0	54 (15.8)	10 (9.5)	
N1	213 (62.3)	73 (69.5)	
N2	57 (16.7)	17 (16.2)	
N3	18 (5.3)	5 (4.8)	
Overall stage^a^			0.690
III	262 (76.6)	83 (79.0)	
IVA-B	80 (23.4)	22 (21.0)	
Schedule dose			0.013
68 Gy/30 F	197 (57.6)	46 (43.8)	
70 Gy/33 F	145 (42.4)	59 (56.2)	

Abbreviations: *RTI* radiotherapy interruption

*P-value calculated by the Chi-square test

^a^According to the American Joint Committee on Cancer, 7th edition

### Survival outcomes

Overall, 342 (76.5%) patients finished their prescribed course of radiotherapy within 5 days of the scheduled time (range: 0–5 days), and 105 (23.5%) patients finished more than 5 days after the scheduled time (range: 6–29 days). The median follow-up was 59.8 months (range: 1.3–76.4 months). At their final follow-up visit, 95 patients had treatment failure because of local relapse (*n* = 28), nodal relapse (*n* = 15) or development of distant metastasis (*n* = 58). Six patients (1.3%) suffered at least two types of treatment failure and 64 patients (14.3%) did not survive. Salvage local treatment included nasopharyngectomy, chemotherapy or re-irradiation. In addition, 9 patients in the group of RTI ≤ 5 days and 16 patients in the group of RTI >  5 days received further treatment for local relapse, but this difference was not significant (*P* = 0.645).

**Fig. 2:**
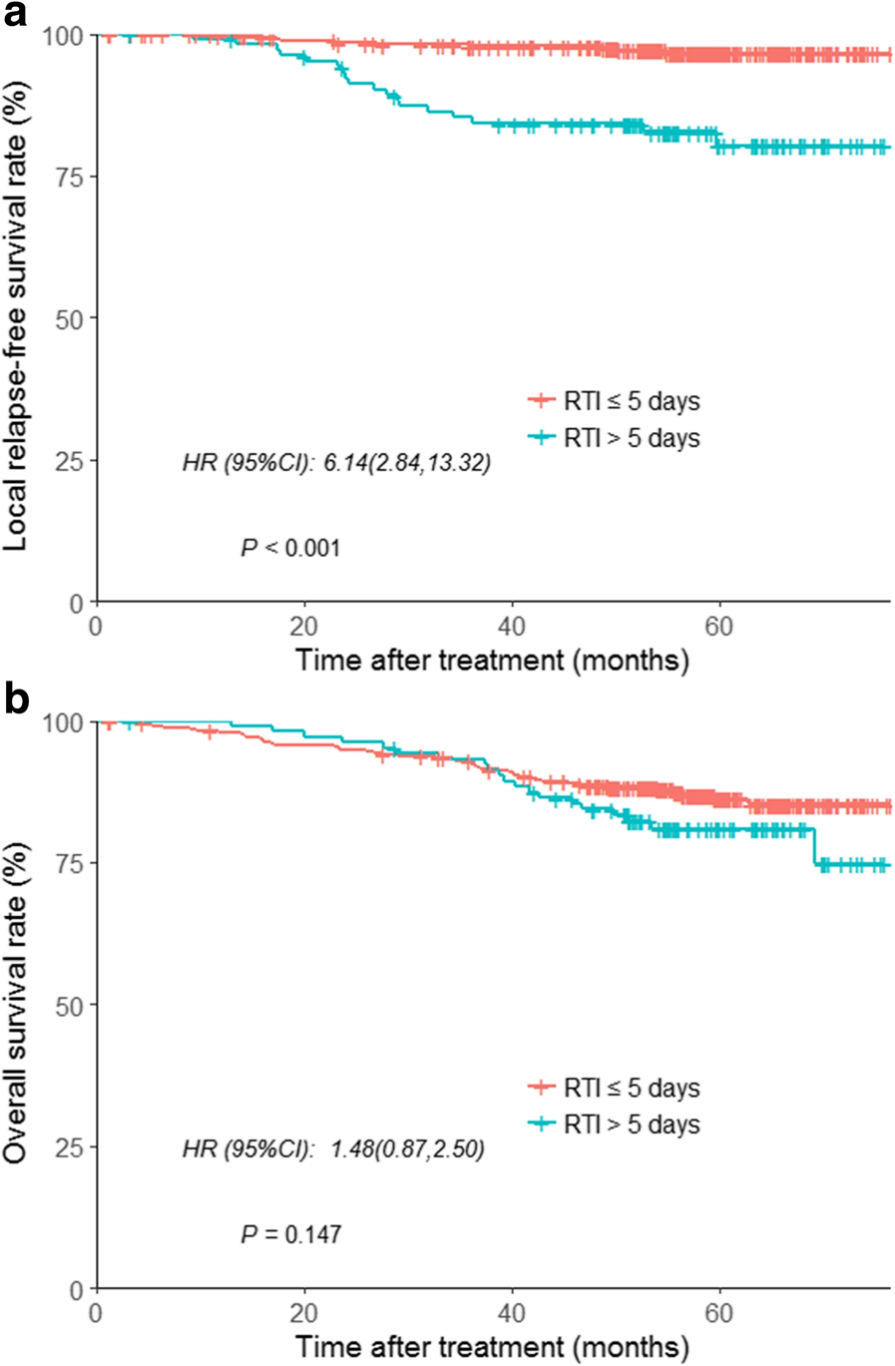
Kaplan–Meier curves for the entire patients stratified by RTI (≤5 vs > 5 days). **a** Local relapse-free survival, and **b** overall survival

Overall, the 5-year LRFS and OS rates were 93.7 and 85.7%, respectively. The 5-year LRFS of the RTI ≤ 5 days group and RTI >  5 days group were 97.1 and 82.9% respectively, a significant difference (*P* < 0.001, Fig. 2a). However, the 5-year OS rates were almost identical in both groups (RTI ≤ 5 vs >  5 days group: 87.1% vs 81.0%; *P* = 0.147, Fig. 2b). The 5-year LRFS rates for the 68 Gy/30F group and 70 Gy/33F groups were 94.0 and 93.3%, respectively (*P* = 0.962). The 5-year OS rates for the 68 Gy/30F group and 70 Gy/33F groups were also similar (85.6% vs 84.5%; *P* = 0.942).

### Prognostic factors

Univariate analysis showed that T stage, overall stage and RTI were prognostic factors for LRFS; OS were significantly associated with age, N stage, T stage and overall stage (*P* < 0.05 for all; Table 2). In multivariate analysis, following parameters as variables were included: age (≤ 50 vs. > 50 years), sex (male vs. female), pathology (type I/II vs. type III), T stage (T3 vs. T4), N stage (N0–1 vs. N2–3), overall stage (III vs. IVA-B) and schedule dose (68 Gy/30 F vs. 70 Gy/33 F). The outcomes for LRFS and OS are presented in Table 3. Significant predictors of inferior OS included age >  50 years (HR = 2.06; 95% CI, 1.24–3.44), N2/3 nodal stage (HR = 1.99; 95% CI, 1.13–3.52) and stage IVA-B (HR = 2.64; 95% CI, 1.07–6.56). Only RTI > 5 days (HR = 9.64, 95% CI = 4.10–22.65) was significantly associated with inferior local control in multivariate analysis.

**Table 2:** Univariate analysis for LRFS and OS

Endpoints	Characteristic	HR	95% CI
LRFS			
	Age (≤ 50 vs. > 50)	1.53	0.73–3.24
	Sex (male vs. female)	1.24	0.55–2.82
	T stage (T3 vs. T4)	2.23	1.01–4.92
	N stage (N0–1 vs. N2–3)	1.36	0.58–3.19
	Overall stage (III vs. IVA-B)	2.45	1.15–5.22
	RTI (≤ 5 vs. > 5 days)	6.14	2.84–13.22
	Schedule (68 Gy/30 F vs. 70 Gy/33 F)	1.99	0.90–4.40
OS			
	Age (≤ 50 vs. > 50)	2.03	1.22–3.38
	Sex (male vs. female)	0.56	0.28–1.09
	T stage (T3 vs. T4)	2.09	1.23–3.54
	N stage (N0–1 vs. N2–3)	2.42	1.46–4.02
	Overall stage (III vs. IVA-B)	2.72	1.65–4.46
	RTI (≤ 5 vs. > 5 days)	1.48	0.87–2.50
	Schedule (68 Gy/30 F vs. 70 Gy/33 F)	1.29	0.72–2.31

Abbreviations: *LRFS* local relapse free survival, *OS* overall survival, *HR* hazard ratio, *CI* confidence interval, *RTI* radiotherapy interruption

**Table 3:** Summary of multivariate cox proportional hazards models for LRFS and OS

Endpoints	Characteristic	HR	95% CI
LRFS			
	Age (≤ 50 vs. > 50)	1.63	0.79–3.12
	T stage (T3 vs. T4)	0.72	0.15–3.34
	Overall stage (III vs. IVA-B)	4.01	0.91–17.68
	RTI (≤ 5 vs. > 5 days)	9.64	4.10–22.65
	Schedule (68 Gy/30 F vs. 70 Gy/33 F)	2.03	0.78–8.67
OS			
	Age (≤ 50 vs. > 50)	2.06	1.24–3.44
	Sex (male vs. female)	0.54	0.28–1.07
	T stage (T3 vs. T4)	0.89	0.34–2.30
	N stage (N0–1 vs. N2–3)	1.99	1.13–3.52
	Overall stage (III vs. IVA-B)	2.64	1.07–6.56

Abbreviations: *LRFS* local relapse free survival, *OS* overall survival, *HR* hazard ratio, *CI* confident interval, *RTI* radiotherapy interruption

### The effect of RTI on different T stages

**Fig. 3:**
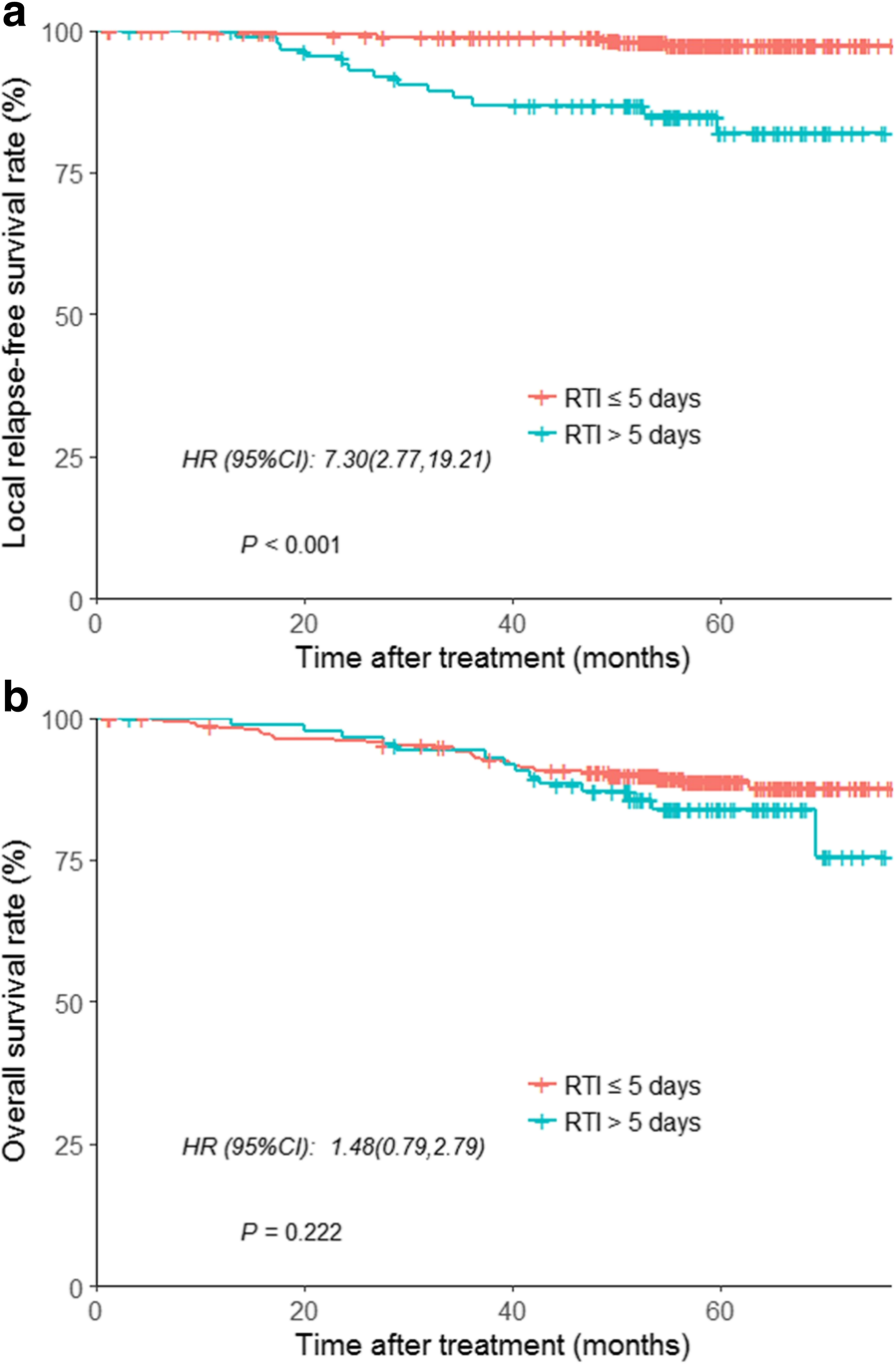
Kaplan–Meier curves for patients with T3 NPC stratified by RTI (≤5 vs > 5 days). **a** Local relapse-free survival, and **b** overall survival

**Fig. 4:**
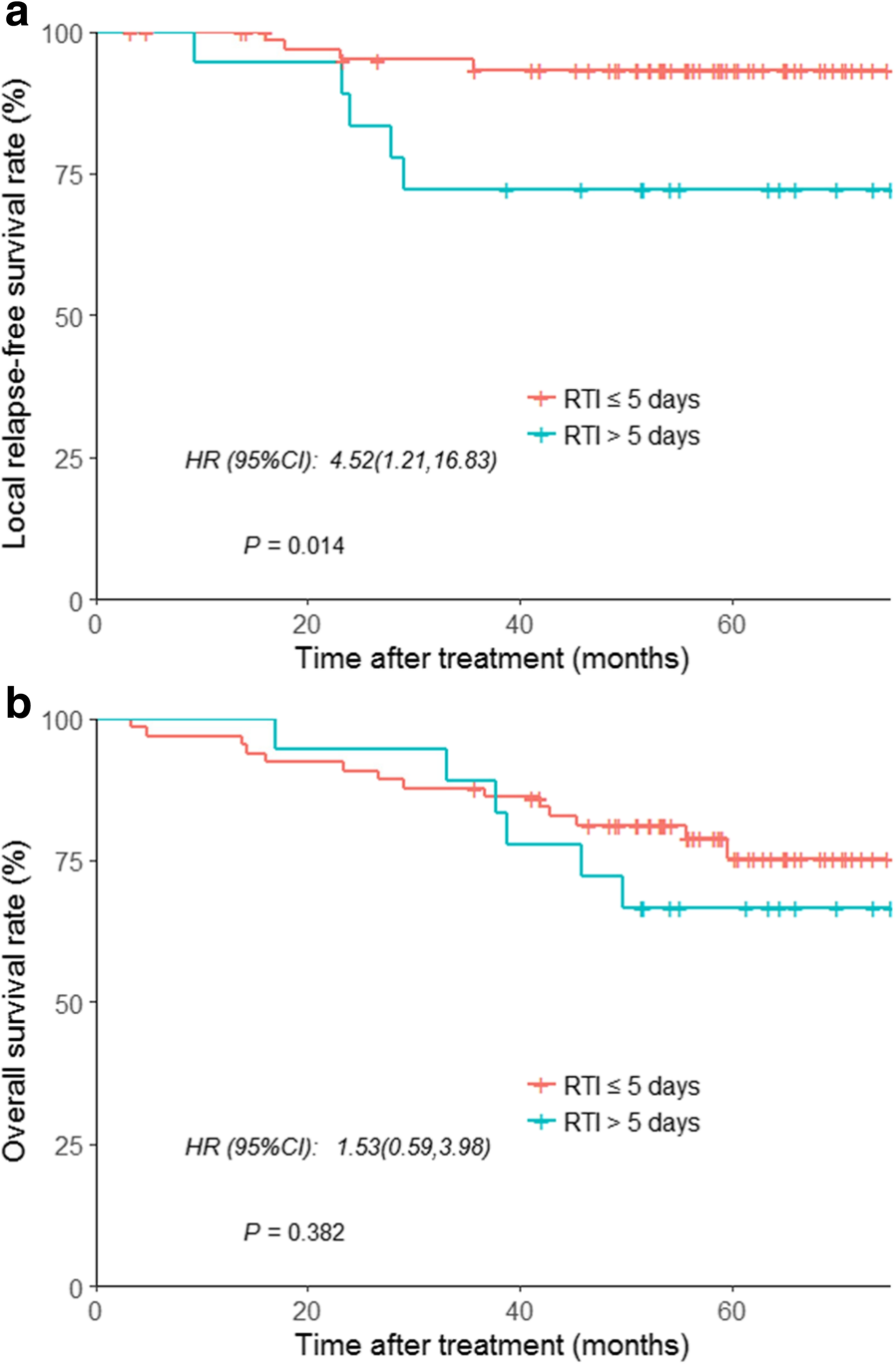
Kaplan–Meier curves for patients with T4 NPC stratified by RTI (≤5 vs > 5 days). **a** Local relapse-free survival, and **b** overall survival

Although no association was found between local control and T stage in multivariate analysis, the Kaplan-Meier model showed a significantly higher risk of local failure for T3 and T4 disease (94.8% vs. 89.2%, respectively; *P* = 0.042). In patients with T3 disease, the 5-year LRFS rates for patients with a RTI ≤ 5 vs. > 5 days were 97.4% vs. 82.1% (HR = 7.30; 95% CI, 2.77–19.21; *P* < 0.001; Fig. 3a). In patients with T4 disease, the 5-year LRFS rates for patients with a RTI ≤ 5 vs. > 5 days were 93.3% vs. 72.2% (HR = 4.52; 95% CI, 1.21–16.83; *P* = 0.014; Fig. 4a). Moreover, in patients with T3 disease, the 5-year rate of OS was 88.9% in the group of RTI ≤ 5 days and 84.1% in the group of RTI > 5 days (HR = 1.48; 95% CI, 0.79–2.79; *P* = 0.222; Fig. 3b) and for T4 stage the rates were 77.9 and 68.7%, respectively (HR = 1.53, 95% CI, 0.59–3.98; *P* = 0.382; Fig. 4b).

### The effect of median RTI in patients with advanced T stage

The median RTI was 3 days (interquartile range: 1–7 days) for the entire cohort. Based on the cutoff point of median RTI, patients were divided into RTI ≤ 3 days group or RTI > 3 days group. Kaplan-Meier method estimates of survival based on the median threshold are shown in Additional file 2: Figure S1. In the log-rank test, RTI > 3 days was associated with inferior LRFS (HR, 4.14; 95% CI, 1.76–9.73; Additional file 2: Figure S1a). However, we did not observe any difference in OS between patients with RTI > 3 and RTI ≤ 3 days (85.0% vs 85.0%; *P* = 0.863; Additional file 2: Figure S1b). Thus, compared with OS, LRFS is potentially more likely to be impacted by RTI. After adjusting for the TNM stage and other variables, we failed to detect an association between RTI (HR, 3.64; 95% CI, 0.97–8.96) and LRFS. In contrast, we found age (HR, 2.06; 95% CI, 1.24–3.44), N stage (HR, 1.99; 95% CI, 1.13–3.52), and overall stage (HR, 2.64; 95% CI, 1.07–6.56) were significant prognostic factors for OS (Additional file 3: Table S2).

## Discussion

Local failure is one of the major treatment failures in NPC, especially for patients with T3–4 stage [18, 19]. Several important prognostic factors for local control have been identified, including radiation technique [7, 18], dose per fraction [20], the volume of tumor [21], T stage [22], daily fraction size [22], presence of Epstein–Barr virus (EBV) DNA [23], RTI [13] and chemotherapy schedule [24]. Of all these factors, the volume of tumor was excluded in the current study due to the difficulty of measuring before treatment. Another potentially valuable prognostic factor is plasma EBV DNA, but the the large interlaboratory variability of EBV DNA enables the difficulty to apply in routine clinical practice. For this reason, we did not include.

In this study, all patients were treated with concurrent radiochemotherapy. Daily fraction size was 2.12 Gy or 2.27 Gy for patients with conventional fractionation. Given the relatively homogeneous in radiation technique, daily fraction size, beam energy, and chemotherapy in the current study, we take more attention to the effect of RTI on local control. Based on the ROC analysis, RTI was analyzed as a categorical variable (RTI either ≤5 or > 5 days) in the present study. The 5-year LRFS rate was 97% if radiotherapy was completed within 5 days of schedule, whereas it was only 83% for RTI > 5 days. Further analysis revealed that RTI was a significant prognostic factor for local control in the current study. However, some studies suggest that RTI may be less relevant for IMRT or chemotherapy in head and neck carcinoma [25]. A recent retrospective analysis was conducted for 321 patients with various stages of localized NPC treated with doses ranging from 64 to 74 Gy over a time period of 5 to 9 weeks [26]. The median RTI was 3 days and no relationship was found between survival outcomes and radiation treatment duration. However, this was likely due to a relatively narrow RTI window and analysis of radiotherapy time as a continuous variable.

Although we found that the 5-year OS rate was higher in the RTI ≤ 5 days group than in the RTI > 5 days group, we did not find a significant correlation between RTI and OS (*P* > 0.05). This could be due to a number reasons. First, OS is not only associated with RTI but also associated with age, sex, N stage, and overall stage, as well as the addition of chemotherapy and supportive care [27]. In the present study, all patients received concurrent chemotherapy that may reduce the effect of RTI on OS. Moreover, salvage treatment after initial treatment failure may be influential. Recently, Chen et al. [28] reported a 2-year OS rate of 84.2% in locally relapse NPC using endoscopic nasopharyngectomy. Moreover, re-irradiation and chemotherapy were associated with satisfactory OS for patients with local recurrent disease [29]. This might partially explain the significant difference in LRFS, but not OS for patients with RTI > or ≤ 5 days.

T stage is known to be a prognostic factor of local relapse of NPC patients [30]. However, we did not find any difference between T3 and T4 disease in terms of local control. This is consistent with a previous study [31], which indicates that the current T-stage does not fully reflect local control in NPC patients after IMRT treatment in combination with chemotherapy. It is well recognized that serious acute side effects that could cause radiotherapy interruption, which have been confirmed to be highly detrimental in radiobiologic efficacy [32, 33]. In this study, we included patients with advanced T-stage, who were more likely to receive a higher radiation dose (> 69 Gy) in combination with a higher intensity of chemotherapy, and the incidence of serious acute side effects could be increased for this group of patients. Moreover, we found patients older than 50 years of age were generally more associated with prolonging RTI. Considering that older patients were more likely to have poor performance status, multiple comorbidities, and inadequate social support, our findings seem reasonable due to patients of older age might have a lower tolerance to intense treatment (RT and/or chemotherapy) [34].

An interesting finding of this study was that patients have a significant difference in distribution of RTI (RTI > or ≤ 5 days) when treated with different fraction size (70 Gy/33 F vs. 68 Gy/30 F). Although we did not observe a significant effect of fraction schedule on survival outcomes, patients treated with 70 Gy/33 F tended to have a longer RTI than patients treated with 68 Gy/30 F. One possible reason might be that patients with 70 Gy/33 F had a longer radiotherapy time in comparison with those treated with 68 Gy/30 F, and they were more likely to experience interruption due to severe acute toxicity, holidays, equipment failure, and other causes.

There are some limitations must be noted. First, the 5-year OS curves were not well defined in the groups of RTI ≤ 5 days and RTI > 5 days. The differences in OS between the two groups may be greater with larger sample size. Second, we failed to include data regarding other prognostic factors, such as the alcohol and/or smoking consumption status. However, no studies to date have demonstrated the effect of alcohol consumption or cigarette smoking on local control for NPC.

## Conclusions

In this study, we described the long-term outcomes for patients with T3–4 stage NPC treated with definitive chemoradiotherapy in the IMRT era. Our results suggest that prolonged RTI > 5 days is an independent adverse prognostic factor on local control for this group of patients. We consider that attention to RTI seems to be warranted for patients with advanced T3–4 stage NPC.

## Additional files

Additional file 1: **Table S1.** Patient characteristics. (DOC 26 kb)

Additional file 2: **Figure S1.** Kaplan–Meier curves for patients with NPC patients stratified by RTI (≤3 vs > 3 days). (A) Local relapse-free survival, and (B) overall survival. (JPG 349 kb)

Additional file 3: **Table S2.** Univariate and multivariate analysis of prognostic factors for LRFS and OS. (DOC 35 kb)

## Abbreviations

2DRT: two-dimensional radiotherapy
AJCC: American Joint Committee on Cancer
CI: confidence intervals
GTV-N: nodal gross tumor volume
GTV-P: primary gross tumor volume
HR: hazard ratios
IMRT: intensity-modulated radiation therapy
LRFS: local relapse-free survival
MRI: magnetic resonance imaging
NPC: nasopharyngeal carcinoma
OS: overall survival
PTV: planned target volume
ROC: receiver operating characteristic
RTI: radiotherapy interruption
