# Supplementary material for: The detrimental effects of radiotherapy interruption on local control after concurrent chemoradiotherapy for advanced T-stage nasopharyngeal carcinoma: an observational, prospective analysis
Source: BMC Cancer. 2018 Jul 16;18:740. doi: 10.1186/s12885-018-4495-2 (PMC6048841; doi:10.1186/s12885-018-4495-2)
Supplement: Supplementary file 1 — Table S1. Patient characteristics. (DOC 26 kb) [file 12885_2018_4495_MOESM1_ESM.doc]

**Table S1.** Patient characteristics

| Age, median (range), years |  | 45 (19-70) |
| --- | --- | --- |
| Sex, No. (%) |  |  |
| Male |  | 338 (75.6) |
| Female |  | 109 (24.4) |
| Pathology, No. (%) |  |  |
| I |  | 2 (0.4) |
| II |  | 20 (4.5) |
| III |  | 425 (95.1) |
| T stage^§^, No. (%) |  |  |
| T3 |  | 364 (81.4) |
| T4 |  | 83 (18.6) |
| N stage^§^, No. (%) |  |  |
| N0 |  | 64 (14.3) |
| N1 |  | 286 (64.0) |
| N2 |  | 74 (16.6) |
| N3 |  | 23 (5.1) |
| Overall stage^§^, No. (%) |  |  |
| III |  | 345 (77.2) |
| IVA-B |  | 102 (22.8) |
| Fraction schedule, No. (%) |  |  |
| 68 Gy/30 F |  | 243 (54.4) |
| 70 Gy/33 F |  | 204 (45.6) |

§According to the American Joint Committee on Cancer, 7th edition.
