# Supplementary figures and images for: The detrimental effects of radiotherapy interruption on local control after concurrent chemoradiotherapy for advanced T-stage nasopharyngeal carcinoma: an observational, prospective analysis
Source: BMC Cancer. 2018 Jul 16;18:740. doi: 10.1186/s12885-018-4495-2 (PMC6048841; doi:10.1186/s12885-018-4495-2)

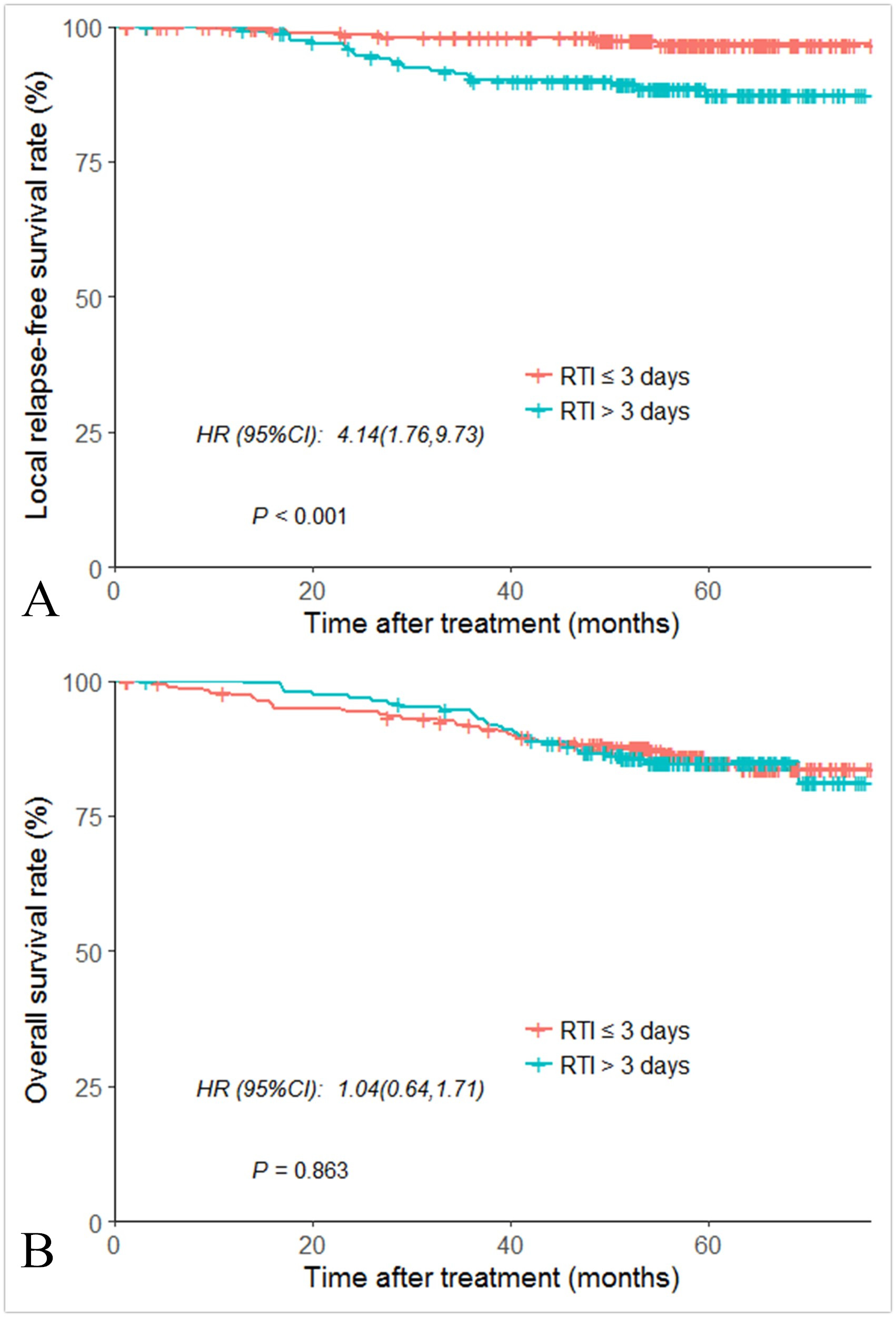

Supplement: Supplementary file 2 — Figure S1. Kaplan–Meier curves for patients with NPC patients stratified by RTI (≤3 vs > 3 days). (A) Local relapse-free survival, and (B) overall survival. (JPG 349 kb) [file 12885_2018_4495_MOESM2_ESM.jpg]
