# Supplementary material for: The detrimental effects of radiotherapy interruption on local control after concurrent chemoradiotherapy for advanced T-stage nasopharyngeal carcinoma: an observational, prospective analysis
Source: BMC Cancer. 2018 Jul 16;18:740. doi: 10.1186/s12885-018-4495-2 (PMC6048841; doi:10.1186/s12885-018-4495-2)
Supplement: Supplementary file 3 — Table S2. Univariate and multivariate analysis of prognostic factors for LRFS and OS. (DOC 35 kb) [file 12885_2018_4495_MOESM3_ESM.doc]

**Table S2.** Univariate and multivariate analysis of prognostic factors for LRFS and OS.

|  | **Univariate analysis** | |  | **Multivariate analysis** | |
| --- | --- | --- | --- | --- | --- |
| **Variable** | HR | 95% CI |  | HR | 95% CI |
| **LRFS** |  |  |  |  |  |
| Age (< 60 vs. ≥ 60 ) | 1.53 | 0.73-3.24 |  | NA | NA |
| Sex (male vs. female) | 1.24 | 0.55-2.82 |  | NA | NA |
| T stage (T3 vs. T4) | 2.23 | 1.01-4.92 |  | 0.76 | 0.16-3.56 |
| N stage (N0-1 vs. N2-3) | 1.36 | 0.58-3.19 |  | NA |  |
| Overall stage (III vs. IV) | 2.45 | 1.15-5.22 |  | 3.33 | 0.76-14.50 |
| RTI (≤ 3 vs. > 3 days) | 4.14 | 1.76-9.73 |  | 3.64 | 0.97-8.96 |
| Schedule (68 Gy/30 F vs. 70 Gy/33 F) | 1.26 | 0.96-1.64 |  | 2.26 | 0.81-5.04 |
| **OS** |  |  |  |  |  |
| Age (< 60 vs. ≥ 60 ) | 2.03 | 1.22-3.38 |  | 2.06 | 1.24-3.44 |
| Sex (male vs. female) | 0.56 | 0.28-1.09 |  | 0.54 | 0.28-1.07 |
| T stage (T3 vs. T4) | 2.09 | 1.23-3.54 |  | 0.89 | 0.34-2.30 |
| N stage (N0-1 vs. N2-3) | 2.42 | 1.46-4.02 |  | 1.99 | 1.13-3.52 |
| Overall stage (III vs. IV) | 2.72 | 1.65-4.46 |  | 2.64 | 1.07-6.56 |
| RTI (≤ 3 vs. > 3 days) | 1.04 | 0.64-1.71 |  | NA | NA |
| Schedule (68 Gy/30 F vs. 70 Gy/33 F) | 1.09 | 0.90-1.32 |  | NA | NA |

Abbreviations: LRFS: local relapse free survival; OS: overall survival; HR: hazard ratio; CI: confidence interval; RTI: radiotherapy interruption; NA, not available.
